# Dental disease and dietary isotopes of individuals from St Gertrude Church cemetery, Riga, Latvia

**DOI:** 10.1371/journal.pone.0191757

**Published:** 2018-01-24

**Authors:** Elina Petersone-Gordina, Charlotte Roberts, Andrew R. Millard, Janet Montgomery, Guntis Gerhards

**Affiliations:** 1 Department of Archaeology, Durham University, Durham, United Kingdom; 2 Institute of Latvian History, University of Latvia, Riga, Latvia; Institute for Anthropological Research, CROATIA

## Abstract

This research explores oral health indicators and stable carbon and nitrogen isotope data to explore diet, and differences in diet, between people buried in the four different contexts of the St Gertrude Church cemetery (15^th^– 17^th^ centuries AD): the general cemetery, two mass graves, and a collective mass burial pit within the general cemetery. The main aim is to assess whether people buried in the mass graves were rural immigrants, or if they were more likely to be the victims of plague (or another epidemic) who lived in Riga and its suburbs. The data produced (from dental disease assessments and isotope analyses) were compared within, as well as between, the contexts. Most differences emerged when comparing the prevalence rates of dental diseases and other oral health indicators in males and females between the contexts, while isotope analysis revealed more individual, rather than context-specific, differences. The data suggested that the populations buried in the mass graves were different from those buried in the general cemetery, and support the theory that rural immigrants were buried in both mass graves. Significant differences were observed in some aspects of the data between the mass graves, however, possibly indicating that the people buried in them do not represent the same community.

## Introduction

**Fig 1:**
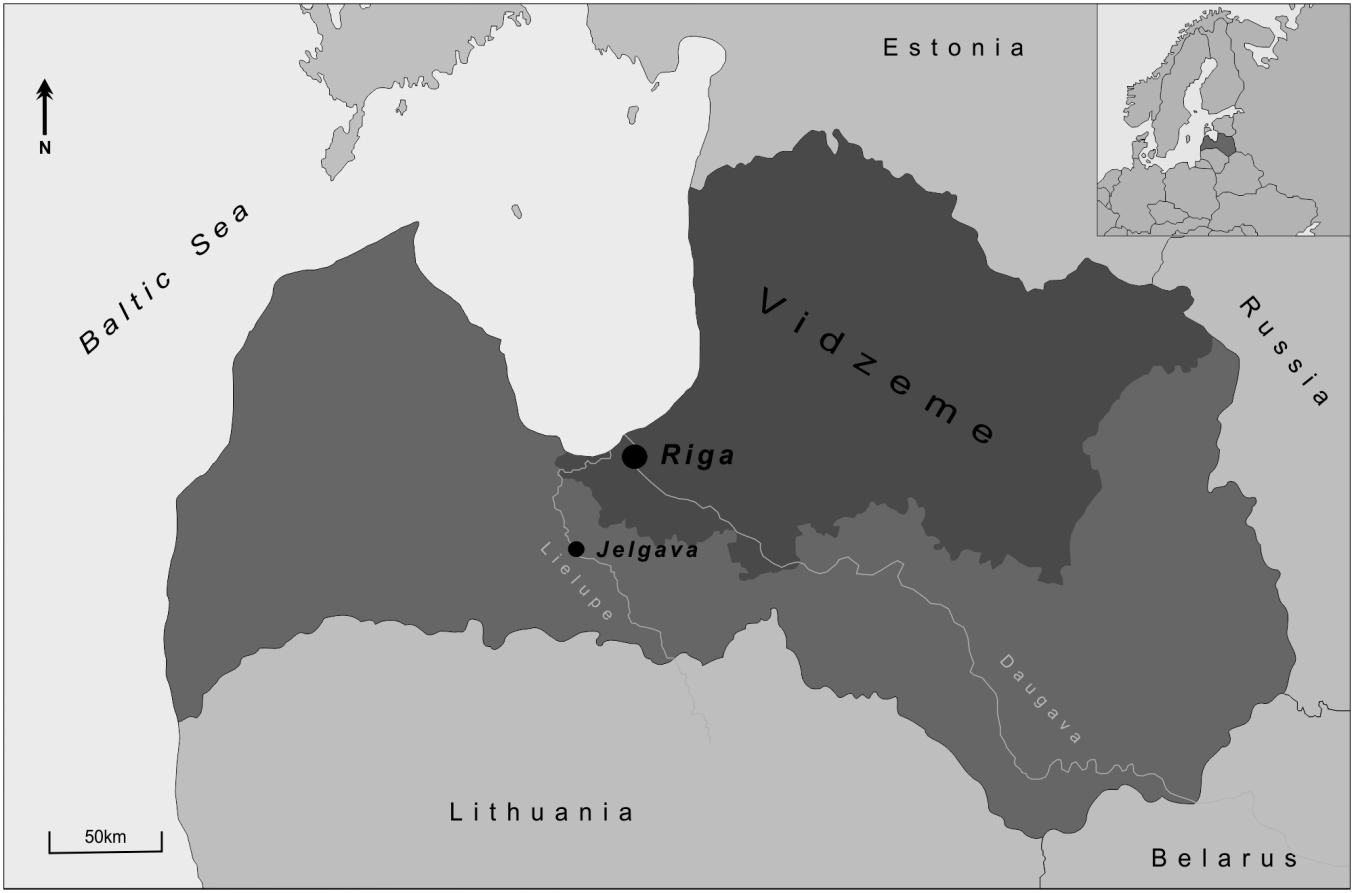
Map of Latvia.

**Fig 2:**
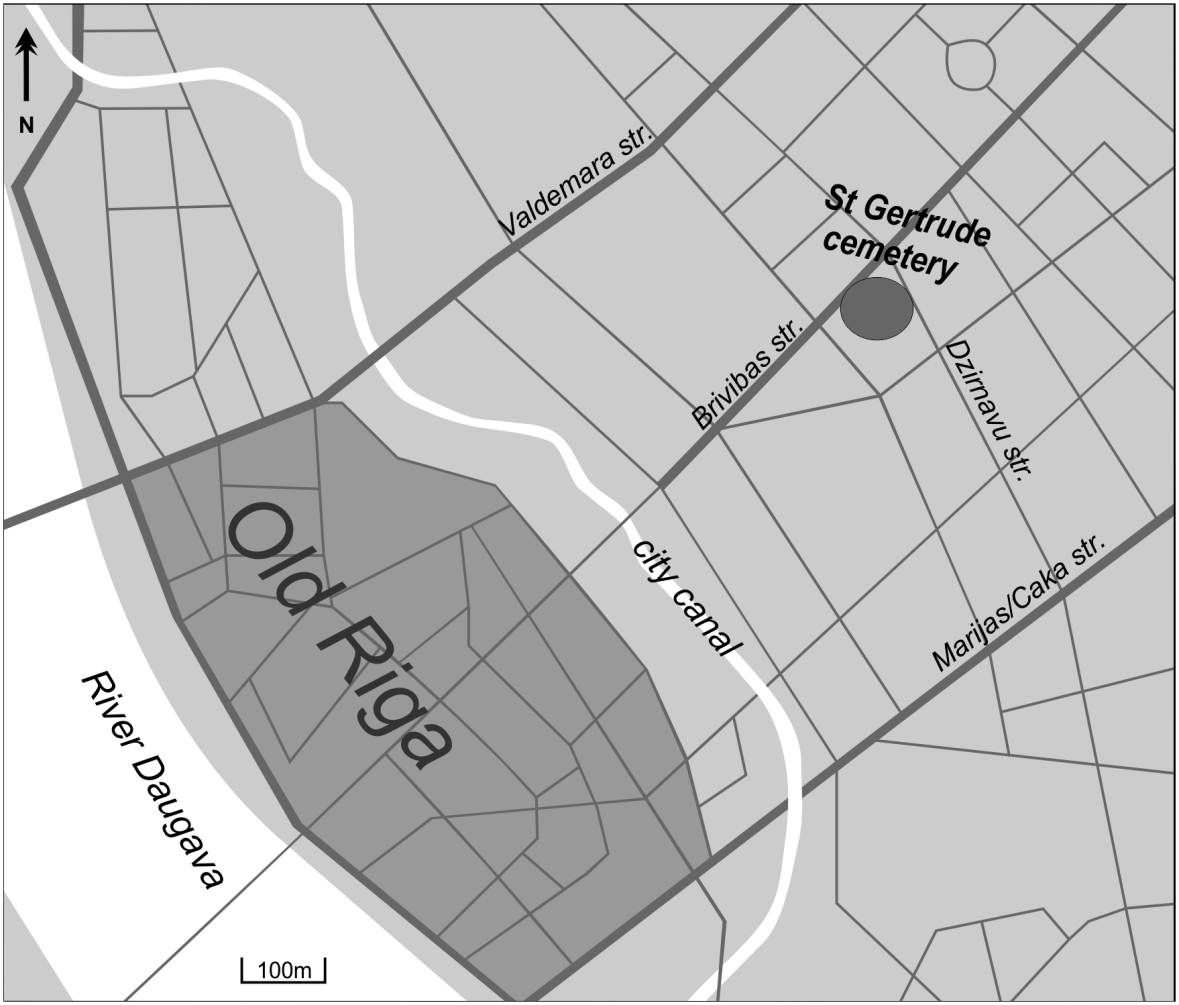
Location of St Gertrude Church cemetery in Riga.

This study focuses on the skeletons excavated from the St Gertrude Church cemetery, Riga, Latvia, dating from the late 15^th^– 17^th^ centuries AD (Figs 1 and 2). The church was located outside the old city wall during the period of its use, and mainly serviced Gertrude village [1]. Although it is believed that the Gertrude village population was moderately wealthy and lived in less crowded conditions than the inner-city population, historical evidence suggests that the suburbs of Riga were destroyed three times between the 15^th^ and 17^th^ centuries by the authorities of Riga ahead of invading armies [2]. Moreover, during the excavation, two mass graves and a smaller mass burial pit were discovered within the general cemetery. Historical evidence suggests that the people buried in these mass burial sites could have been either the victims of an epidemic or rural immigrants who came to Riga during a devastating famine at the beginning of the 17^th^ century [1, 3, 4].

This research aims to explore the diet of the people buried in the general cemetery and mass graves, by comparing the prevalence of dental disease, calculus, as well as attrition scores in all individuals, and dietary isotope profiles in the adult population, to identify if there were differences in the people interred in the different burial complexes (the general cemetery, two mass graves, and burial pit). Since dental attrition, caries, periodontal disease, and calculus are important indicators of diet, these conditions are used here to reconstruct diet and general dental health. A comparative analysis of oral health indicators between the burial contexts will further confirm whether there is variation in frequencies that indicate different population groups. Data on oral health indicators are complemented by carbon (δ^13^C) and nitrogen (δ^15^N) isotope analyses, which provide insights into the dietary status of the population. The three hypotheses proposed are:

1. Most individuals buried in the general cemetery were from the local area and thus represent a moderately wealthy, urban, population with access to softer dietary carbohydrates, such as finely ground bread, and foods that are accepted as linked to higher status, such as meat. This is predicted to be expressed in a higher prevalence of “destructive” dental diseases (caries, periodontal disease), lower dental attrition rates, and higher δ^15^N values, indicative of more animal protein in their diet;
2. Most individuals buried in one, or both, mass graves represent poor rural immigrants. This is predicted to be expressed in a lower prevalence of destructive dental diseases, higher dental attrition rates, and lower δ^15^N values, indicating a diet dominated by plants;
3. Most individuals buried in one, or both, mass graves represent victims of plague, or another epidemic disease, who lived in Riga and/or its vicinity. This scenario is predicted to be expressed in similar attrition scores and prevalence rates of dental pathological conditions and δ^13^C and δ^15^N values, in line with people from the general cemetery.

### Historical background and a comparison of living conditions in urban and rural areas during the post-medieval period

St Gertrude church was built around 1413 AD outside the old centre of Riga, to accommodate the city’s growing population and to provide shelter for travellers. The church was located on the main route to the east, leading to the Vidzeme region, of which Riga is the capital, and further afield to Estonia and Russia [1].

St Gertrude’s Cemetery mostly received the local, moderately wealthy suburban community from Gertrude village and its vicinity [1], but the cemetery has also been mentioned in historical sources as the final resting place for other population groups and plague victims [3, 4]. Notably, in the winter of 1601–2, poor immigrants from rural Vidzeme flocked to Riga in search of food, but were forced to camp outside the city walls near St Gertrude church, where they died in great numbers from hunger and cold; the deceased were most likely buried in the cemetery [1, 3, 4]. The presence of mass graves in the cemetery supports the historical evidence and suggests that people buried there could represent a different, possibly poor rural, population. Alternatively, victims of the plague from Riga and its suburbs could also be present in the mass graves, since St Gertrude graveyard is mentioned in historical sources as one of the burial places for 17^th^ century plague victims [1]. There were at least two plague epidemics in Riga, in 1601 and 1623, according to Bodecker’s chronicle [5].

The population of Gertrude village mostly comprised peasants and craftsmen [6]. There is evidence that the peasants of Riga and its suburbs had increased freedom from their landowners, compared to most peasants from rural areas [2]. The status of Riga as a key trading centre provided them with direct access to resources arriving into the city, possibly including goods from newly acquired European colonies in Africa and America, such as sugar [7]. There is also evidence for numerous beehive trees near Gertrude village, and it is possible that villagers could use this resource in their diet, especially if they were involved in honey harvesting [6]. The better social status of peasants from Riga, combined with accessibility to Riga’s markets, might well have had a positive effect on the quality of the diet of these people, as expressed in more variation in dietary sources, and probably more animal protein in general, compared to that available to the poor rural populations (see below). Although the suburban population of Riga benefited from less crowded living conditions than within the city walls, they were much less protected than the city population, especially during warfare. Most buildings were constructed from wood to allow the authorities to burn down the suburban areas ahead of sieges. This was done to destroy potential shelter and resources for the invading army. The suburbs of Riga were thus deliberately destroyed in 1559, 1605 and 1710. The city also allegedly suffered from several famines and plague epidemics in the 16^th^ to 18^th^ centuries, mainly due to frequent conflicts [2]. According to historical sources, such disasters affected the availability of resources for all social groups regardless of status and wealth throughout the region, and thus could have affected the health status of the living population at least to some extent. This was the case for a wealthy post-medieval German population buried in Jelgava, Latvia, where a recent study found evidence for nutritional stress in children, as expressed in high rates of cribra orbitalia and scurvy. The presence of these conditions in non-adult skeletons, combined with high rates of linear enamel hypoplasia in adult individuals, was interpreted as evidence for possible hardships during frequent wars, famines and epidemics during the lifetimes of these people [8].

With regard to the rural peasant population, it is believed that their living conditions were poorer than those of the urban population in most regions of Latvia. The majority of people in the countryside were peasants, who were the subjects of German landowners. These people had to work in the landowners’ farms to make them profitable, but they were not allowed to use the produce of the farm for subsistence; as a result, they had to work in their own allotments after they returned from work on the farm, in order to feed themselves and their families. Due to the limited size of the allotments, as well as minimal input of work, there was rarely any leftover produce to sell in the markets, and transporting the produce to the market would have been difficult for those living in remote areas [9]. An account of the diet of Latvian and Estonian peasants suggests that the quality of meals depended on the season, with the period between Christmas and early summer being the leanest, while salted fish, as well as butter were available in the summer, and meat was eaten on Sundays during the autumn and winter [10]. The availability of these foodstuffs, however, would have differed across regions. Indeed, previous studies on the stature of post-medieval cemetery populations from Latvia have revealed that the lowest statures characterized rural peasant populations from the poorest regions [11]. Stature that is reduced according to the norm for a population from a particular geographic region has been linked to poor health and/or undernutrition in developing countries today [12–14], and in many archaeological populations [15, 16].

### Criteria for the selection of the oral health indicators used in this study, and their brief aetiologies

The dental pathological conditions studied for this research were chosen according to their potential to reveal information about diet and to test the three hypotheses proposed. It was believed that the oral health indicators which are largely related to diet (dental attrition, caries, periodontal disease, periapical lesions, ante-mortem tooth loss, and calculus deposits), would best highlight differences between the individuals buried in the general cemetery and the mass graves. Indeed, many have synergies. The presence and extent of dental attrition, as reflected in skeletal remains from archaeological sites, often depends on the coarseness of the diet, as well as the amount of abrasive particles in the food [17, 18]. Some archaeological studies have linked slight dental wear in combination with high caries rates to soft and sticky foods in the diet, which is taken to be indicative of increased wealth in archaeological populations [19, 20].

Caries formation is a slow and gradual process caused by organic acids, which are known to form in the fermentation process of dietary carbohydrates, and sugars in particular, by plaque bacteria [21–25]. Caries is highly prevalent in industrialised populations, routinely affecting 60–90% of schoolchildren and most adults [26]. It is believed to have increased considerably with the advent of a sedentary lifestyle in the past, since this was consistent with eating an increased proportion of carbohydrates, such as those derived from farmed crops [22, 27]. The introduction of refined sugar from the European colonies to Europe in the post-medieval period caused a rapid deterioration of dental health. Initially only the wealthy had access to sugar, but eventually it became more widely available for the whole population [25]. This has also been shown by high caries rates recorded in a high-status 17^th^ century population from Jelgava, Latvia, who were known to have access to refined sugar [28]. Consequently, significant differences in caries prevalence rates between post-medieval populations might point to differential proportions of carbohydrates in the diet, as well as access to refined sugar. A number of bioarchaeological studies have proved that older individuals and females are generally more likely to develop the lesions [19, 20, 22, 29–34]. Higher caries rates in females in archaeological populations have most commonly been explained by gender differentiation of diets [29] and the demands of pregnancy [22, 29–31, 35]. In addition, some evidence suggests that an increase of oestrogen levels in female saliva during pregnancy could be responsible for higher rates of caries in this sex group [36]. This is supported by a recent clinical study, which showed increased levels of salivary Streptococcus mutans, a type of the bacteria which has been linked to development of caries, in pregnant women [37, 38].

Caries-induced infection of the pulp cavity is believed to be one of the most common reasons for the development of periapical lesions [39–41], and their presence might be indicative of dental decay even when the teeth have been lost ante- or post-mortem. Severe attrition also can lead to infection, once the pulp cavity is exposed [40]. On the other hand, there are several types of periapical lesions, including granulomata and cysts, and not all necessarily become infected during a person’s life [42]. Distinguishing between benign and infected periapical lesions and their causes in archaeological populations can be difficult, and thus their presence cannot be readily taken as evidence for poor dental health [43].

Periodontal disease, like caries, has been found to have a strong relationship to plaque bacteria [44, 45], and therefore the prevalence of both conditions is often similar in archaeological populations [40]; this was also the case in the high-status Jelgava population, mentioned above with regard to high caries rates [28]. Likewise, periodontal disease tends to have a higher prevalence in populations which consume soft, processed foods [46]. Clinical data show that increased levels of psychological stress can exacerbate periodontal disease in populations today [47, 48]. Its main consequence is alveolar bone loss, which is most likely caused by advanced bacterial infection of the gingiva (the soft tissue surrounding the teeth) [49]. In this study, periodontal disease was viewed in relation to caries, as a potential indicator of the consumption of carbohydrates in the diet.

Caries, and especially periodontal disease, are among the leading causes for ante-mortem tooth loss (AMTL) [20, 42, 45]. In archaeological studies on historical populations, AMTL has been viewed in relation to differences in social status, with wealthier individuals often exhibiting better oral health and thus, lower rates of AMTL, compared to people of lower social status; these differences were explained by a higher proportion of protein and a comparatively low amount of carbohydrates in the diet of high-status individuals, as opposed to poorer members of the society [29, 50, 51]. The main aim of including AMTL in this study was to provide an approximate indicator of overall dental health, while the proportion of protein in the diet of people buried in St Gertrude’s cemetery population is estimated by dietary isotope analysis.

Dental plaque is characterized by a film of micro-organisms covering the tooth surface in living people [21, 24]. Mineralised plaque, or calculus, deposits are very common on the teeth of archaeological skeletons [17]. Calculus formation on the teeth depends on various factors such as components in the diet and an inherited predisposition [21]. With regard to diet, it has been found that calculus deposits tend to increase in agricultural populations due to diet dominated by soft, cooked carbohydrates, as opposed to more fibrous food used by hunter-gatherers, which is consistent with changes in the composition of plaque bacteria in response to different dietary practices [46]. On the other hand, recent clinical studies have shown that considerable amounts of an amino-acid, L-arginine, which is naturally found in red meat, poultry and milk, effectively removes dental plaque [52, 53]. It is not currently known, however, if a diet rich in animal protein would also act against plaque bacteria developing. In this study, the presence and amount of calculus is considered with regard to the other plaque-related diseases of caries and periodontal disease.

### A brief overview of carbon and nitrogen isotope analysis

Carbon (δ^13^C) and nitrogen (δ^15^N) isotope analyses of collagen were employed in this research in order to provide a more detailed insight into the diet of the people buried in the cemetery. This method of analysis cannot detect carbohydrates unless protein consumption is extremely low and thus cannot directly support evidence for a high prevalence of destructive dental disease. However, it remains a useful tool in detecting the proportion of animal (δ^15^N) and marine proteins in the diet (δ^13^C). Different δ^13^C and δ^15^N values might help to distinguish if there were:

- groups of people from different regions (closer and further from the coast),
- differential access to animal protein, and/or
- different diets for males and females

Moreover, certain animal (milk, cheese) and marine (fish) proteins have the potential to prevent dental decay [23] and therefore the results of macroscopic and isotopic analyses are cross-compared.

Since the first use of carbon and nitrogen stable isotope analyses in relation to exploring diet in past populations [54–57], they have become one of the most important means of studying dietary choices [58–64]. Isotope analysis is of particular importance in archaeology due to its potential to detect different food sources and, most importantly, how these sources were used by different age, sex and status groups within the same population. In essence, δ^13^C values reflect the presence of food chains based on plants with different photosynthetic pathways in the diet (C_3_ and C_4_ plants, which grow worldwide and mainly in tropical regions, respectively), as well as the contribution of marine resources to the diet [65]. In Northern European studies, especially with regard to periods before the introduction of C_4_ plants such as maize from the 16^th^ century onwards [66], δ^13^C values are mainly used to study the proportion of marine resources in the diet [61, 67]. According to previous studies, a diet based entirely on marine resources will yield δ^13^C values of around -12‰, while a C_3_ diet with minimal or no marine input will result in δ^13^C values around -21‰ [68–72]. In contrast, a diet based on C_4_ plants will yield values of -14‰, or higher [73], and could indicate access to imported grains in post-medieval northern European populations.

The only source of nitrogen in the diet is protein [74, 75]. δ^15^N values reflect the trophic level of living organisms, and therefore they can be used to detect the proportion of animal protein as well as marine or freshwater resources in the human diet [59, 76]. While it is not possible to accurately estimate the percentage of animal protein in the diet, a higher δ^15^N value does indicate a higher proportion of meat or secondary animal proteins, such as milk [59, 77]. Likewise, due to substantially higher δ^15^N values in fish compared to terrestrial resources, the presence of marine or freshwater resources in a diet will also be expressed as higher δ^15^N values in bone collagen samples [78, 79].

## Material

Seven hundred and twenty-one skeletons were excavated from St Gertrude’s cemetery (Latitude 56.954818, Longitude 24.119343) between August and October 2006 prior to planned building works. The archaeological excavation was carried out by Architectural Research Group Ltd (SIA AIG) and supervised by Mārtiņš Lūsēns, with permission No 2006/A-0000466 issued by the State Inspection for Heritage Protection (VKPAI). Since the skeletal remains found during the excavation were older than 100 years, no permissions were required for their research in accordance with the law On the Protection of the Body of Deceased Human Beings and the Use of Human Tissues and Organs in Medicine, 1992 [80]. The excavated skeletal material (collection number 104) is curated at the Institute of Latvian History, University of Latvia, Kalpaka bulv. 4, Riga. The material is accessible for research by prior arrangement in accordance with the Institute’s regulation No 2015/253.

During the excavation, apart from recovering burials from the general cemetery, two mass graves (MG) were discovered in the south-eastern (MG1) and north-western (MG2) corners of the excavated area, containing 166 and 120 individuals, respectively. In total, there were 435 individuals in the general cemetery (GC), including the burial pit (BP), and 286 in the mass graves (Table 1). Individuals in the mass burials were not commingled, and the preservation of their skeletons was very good in all contexts.

**Table 1:** Number of individuals in each context of the cemetery.

*Context*	*Males*	*Females*	*Adults*	*Non-adults*	*Total*
***GC***	115	100	18	187	420
***MG1***	63	48	0	55	166
***MG2***	46	34	0	40	120
***BP***	3	9	0	3	15

In this study, comparative dental pathological and isotope analyses were carried out on the groups from the GC and MGs; the analyses were based on differences in diet, reflected in carbon and nitrogen isotope values, rather than strontium (^87^Sr/^86^Sr ratio) and oxygen (δ^18^O) isotope analyses, which are usually applied to study population origins and mobility [79]. However, a separate pilot study comparing ^87^Sr/^86^Sr ratios in permanent canines from selected non-adult individuals from the cemetery is underway to add to the findings of the current research.

## Methods

### Osteological methods

Sex estimates of adult individuals were based on morphological traits of the pelvic girdle and skull [81–83], with the pelvic data having precedence, if available. Age at death estimates in adults were mainly based on degeneration of the pubic symphysis and auricular surface of the os coxae, using degenerative changes in the sternal ends of the ribs, and cranial suture closure, where this was not possible [84–90]. To estimate age in non-adult individuals, dental formation and eruption were used [91], as well as epiphyseal fusion and long bone measurements [92–95].

Before prevalence analyses were conducted, chi-square statistical significance tests were used to compare the adult age and sex distribution. The distribution of young (18–30 years old) and middle to older individuals (over 31 years old) was not significantly different between the contexts (X^2^ (1, N = 226) = 0.06, p = 0.806). The same was true for the distribution of sex groups for young males and females (X^2^ (2, N = 88) = 0.44, p = 0.802) and older males and females (X^2^ (2, N = 138) = 5.6, p = 0.06). Because the main aim of this research was to compare specific oral health indicators to see if the population groups in each context had different origins, and based on the equal distribution of age and sex groups, the prevalence of dental diseases in men and women is given for both adult age groups together. Data on prevalence for caries, periapical lesions, periodontal disease and ante-mortem tooth loss by age group (18–30 and over 31-year-old individuals) can be found in S1 Table.

Due to the low prevalence of dental disease in non-adult individuals, and to achieve suitable sample sizes, they were not divided into age groups. A statistical test was performed to control for different age structures in each burial context in order to avoid age bias in specific dental conditions, and particularly attrition scores. There was no statistically significant difference in the distribution of children aged between six and 11, and 12 and 17 years who had at least one observable permanent tooth between the contexts (X^2^ (2, N = 83) = 4.99, p = 0.082). Likewise, no statistically significant differences were observed when comparing individuals with at least one observable molar, for attrition analysis (X^2^ (2, N = 67) = 5.66, p = 0.059).

Deciduous and permanent teeth were analysed separately. Otherwise, dental pathological conditions and attrition were recorded similarly in adults and non-adults.

### Palaeopathological methods

Dental attrition was recorded for all present permanent teeth following the diagrams of Murphy [96] and Smith [97]. These methods, which use modal forms rather than individual drawings, were chosen for their simplicity of use and comparability, as well as the reduced possibility of intra- and inter-observer error. The wear on all teeth was recorded in order to control for possible heavy activity-related wear rather than dietary induced wear. Individuals with possible activity-related wear were excluded from this analysis. Teeth affected by severe caries or post-mortem damage were recorded as not observable. The overall attrition for teeth in skeletons from each context was calculated by averaging the wear of the first maxillary and mandibular molars (M1), as suggested by Lunt [98]. The analysis was performed both overall, and according to age group in adults (18–30, and 31+ years old).

The presence of caries was documented for every permanent and deciduous tooth according to Lukacs [99], and recorded as absent or present in every individual with at least one observable tooth. Caries was classed as present if there was a visible lytic lesion penetrating the dental crown or the root of the tooth. Erupting teeth clearly above the alveolar margin were also recorded as observable.

Periapical lesions were recorded as present if a distinctive smooth walled sinus in the alveolar bone was visible at the apex of the root [42]. Every socket with an erupting/erupted tooth was treated as observable, even if the tooth had been lost ante- or post-mortem. The lesions were recorded as present or absent. To avoid recording pseudosinuses as a result of post-mortem damage, the morphology of the sinus walls was observed with a hand lens (12x magnification).

Periodontal disease was assessed and recorded as suggested by Ogden [42] taking into account the morphology of the alveolar margin rather than the length of the exposed root. To avoid intra-observer error, the condition was recorded as absent or present by quadrant without scoring severity (anterior and posterior teeth—incisors and canines, and premolars and molars, respectively). True prevalence rates were also calculated by quadrant. Periodontal disease was recorded as observable even if only one tooth was present. Edentulous individuals were excluded from the analysis.

A tooth was considered as lost ante-mortem if the socket showed signs of remodelling, or had completely remodelled. The presence of calculus was assessed according to Brothwell [17], but to minimise intra-observer error it was scored as slight (1) or medium to heavy (2) on every tooth.

To decide if dental attrition scores were statistically significantly different between different demographic groups and contexts, Kruskal-Wallis and Mann-Whitney tests were used. When comparing three or more groups, a Kruskal-Wallis test was used, followed by a post-hoc Mann-Whitney test if the result was statistically significant. To compare prevalence rates for all other dental diseases, a chi-square test was used on samples larger than five, and a Fisher’s Exact test for smaller samples. In all tests, the significance level was set at 0.05. Only p values are given in the text, but the details of the calculations are given in S2 Table. All osteological and palaeopathological analyses were carried out by one observer, thus excluding any possibility for inter-observer error; to avoid intra-observer error, most lesions were scored as absent and/or present.

### Isotope analysis

Carbon and nitrogen stable isotope analyses were carried out on 96 selected adult individuals from the mass graves (22 samples from MG1 and 23 samples from MG2) and the main cemetery (46 samples), as well as the BP (five samples) to explore possible dietary differences and to complement the findings of the dental analysis. Non-adults were not analysed isotopically in this study. Seven additional samples were analysed from a contemporary Jelgava Holy Trinity Church cemetery population, Latvia, in a different region to see if regional differences between the two cemetery populations were expressed in different δ^13^C and δ^15^N values. These samples were taken from the skeletons of children aged 1–2 years (J15, J22, J33), 2–3 years (J97), 3–4 years (J95, J98), and 4–5 years (J40). For calculations of statistically significant differences in values, a Kruskal-Wallis test was used to compare three or more groups, with a post-hoc Mann-Whitney test if the result was statistically significant. Mann-Whitney test was used to explore comparisons between two groups, with the significance level set at 0.05 for both tests. Linear correlation and regression analysis by Pearson product-moment correlation coefficient was performed on all δ^13^C and δ^15^N values from the St Gertrude population, whereby *r* = +1.0 represents a perfect positive correlation, *r* = −1.0 a perfect negative correlation, while *r* = 0.0 signifies a complete absence of correlation. The purpose of these analyses was to determine if the values were influenced by differing proportions of marine sources in diet [69].

For carbon and nitrogen isotope analysis, 90-200mg of bone were taken from the ribs or the scapulae. These skeletal elements were chosen because they were often already fragmented due to post-mortem damage, thus minimising any new damage to skeletal remains during sample collection. The samples were prepared for collagen extraction, following a modified Longin [100] method [69]. To extract the collagen, the bone samples were demineralised in 0.5M HCI (hydrochloric acid solution) at 4°C for 5–6 weeks. The demineralised samples were rinsed with deionized water and transferred to falcon tubes with a pH3 HCl acid solution, before gelatinisation at 70°C for 48 hours. The samples were then Ezee-filtered, frozen, and freeze-dried.

The prepared collagen samples were measured in duplicate at the School of Archaeological Sciences, University of Bradford, by combustion in a Thermo Flash EA 1112 and introduction of separated N_2_ and CO_2_ to a Delta plus XL via a Conflo III interface. Laboratory and international standards were interspersed throughout each analytical run. The results are expressed using the delta notation in parts per thousand (per mil or ‰) relative to the international VPDB standard for carbon and atmospheric nitrogen for the nitrogen as follows:

$$\delta^{15}N=R_{sample}/R_{standard}-1$$

where *R* is the isotope ratio ^15^N/^14^N or ^13^C/^12^C [101]. The error for the carbon and nitrogen isotope ratio measurements determined from repeated measurement of international and laboratory standards did not exceed +/- 0.2‰, 1 standard deviation. One sample from Jelgava (J40) yielded a higher than recommended C:N ratio (3.6) [102], signalling contamination; this was therefore not used for further analysis. All collagen yields were above 5%.

## Results

### Age and sex distribution

**Fig 3:**
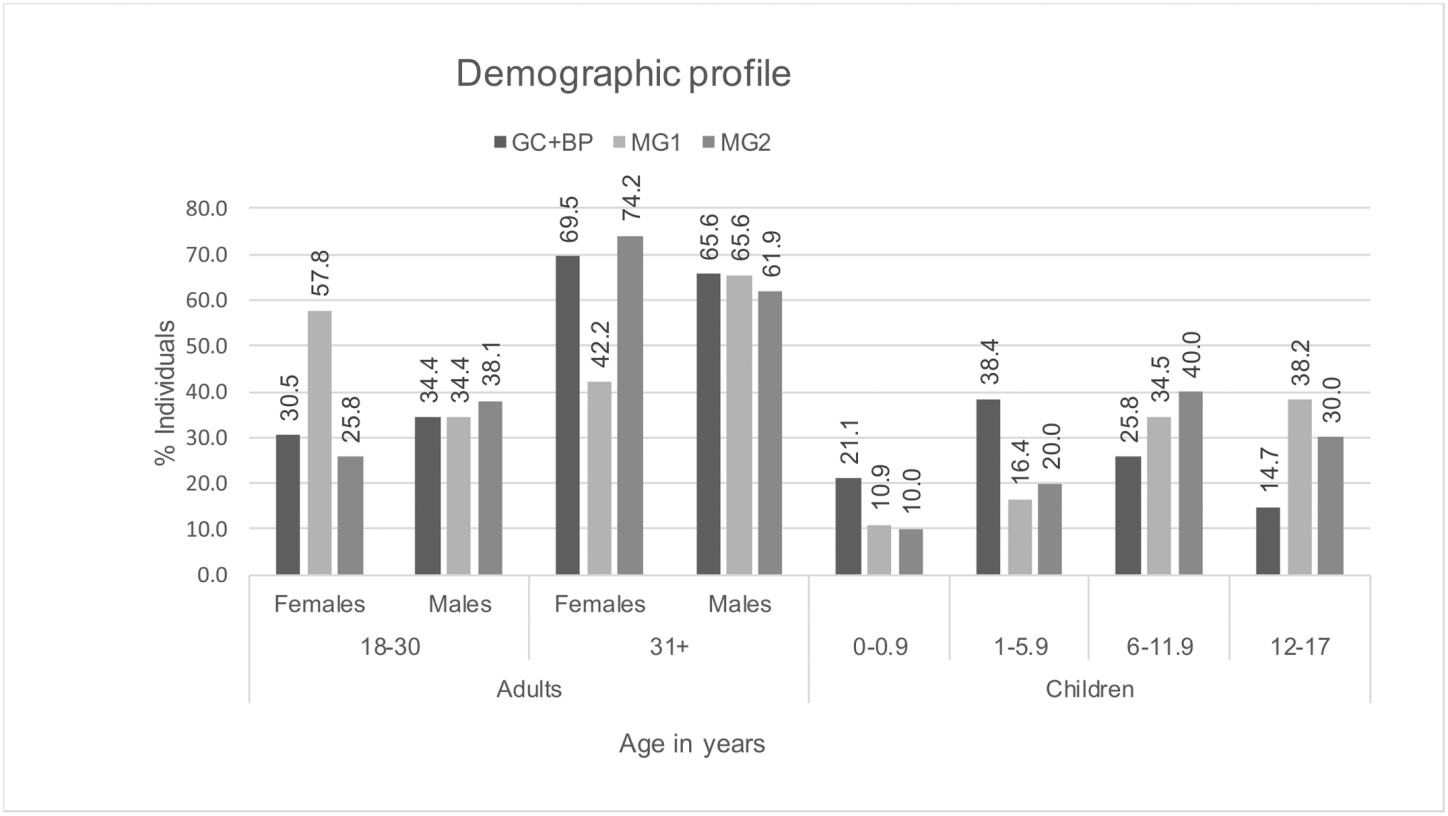
Demographic profile of the population.

The reconstructed demographic profile suggests a catastrophic mortality for those buried in both mass graves [103], whereby most age and sex groups are equally distributed due to being equally susceptible to the cause of death. By contrast, attritional mortality profiles will demonstrate a focus on certain age and sex groups, for example, infants and very young children, and older adults [104]. This was particularly true for the non-adult population, whereby the mass graves contained fewer young individuals, and substantially more older children, than were present in the general cemetery (Fig 3). There were nine females, three males and three non-adult individuals in the BP. All adults were over the age of 30, and the youngest child was 7–8 years old at death. Because of the small sample size, these individuals were combined with the GC population for palaeopathological and isotope analyses.

It was not possible to estimate age for 22 males and 28 females from the GC, two males and three females from MG1, and four males and three females from MG2, and therefore these individuals were not included in the demographic profile.

### Oral health indicators

#### Attrition of M1

**Fig 4:**
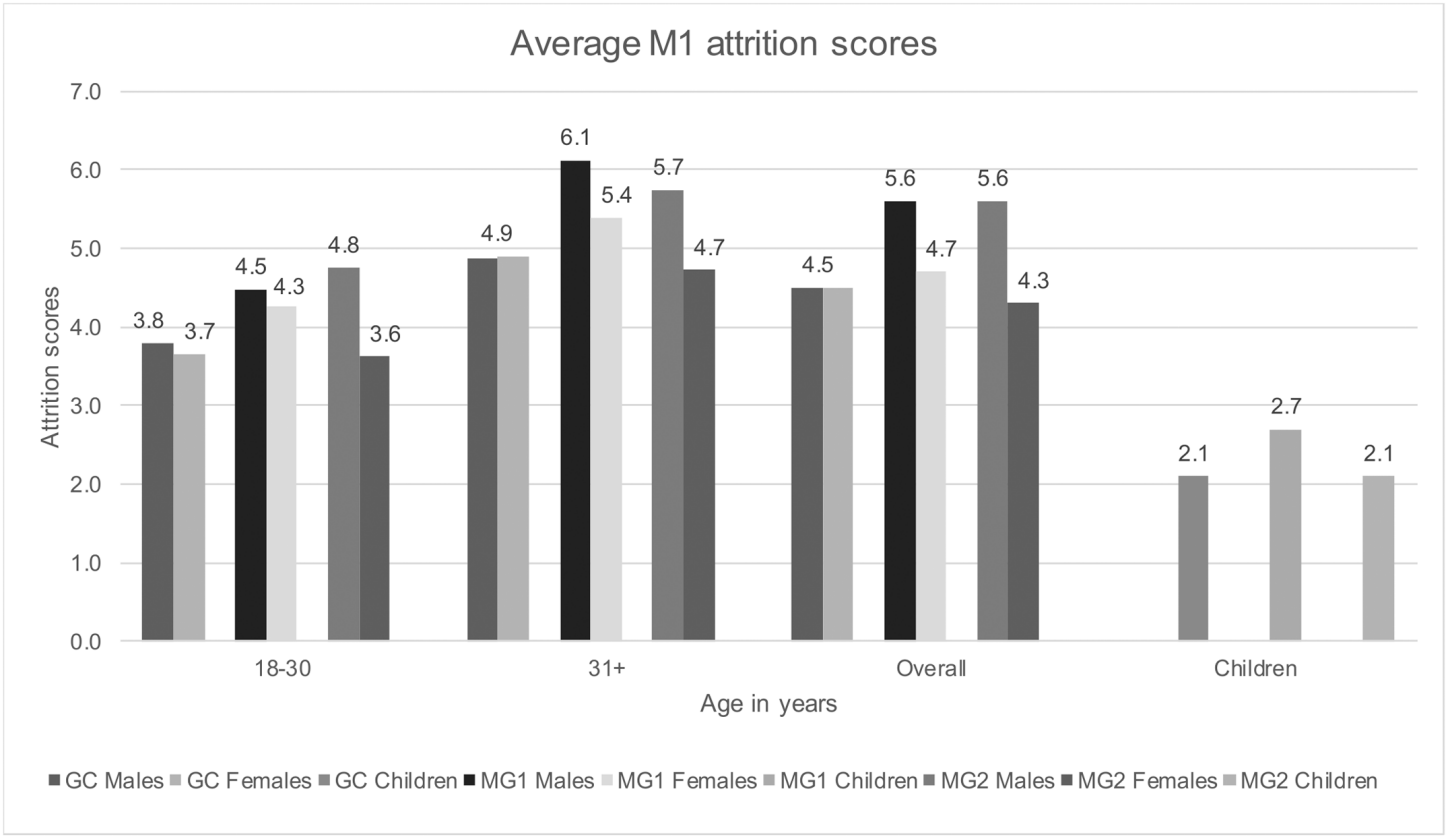
Average M1 attrition scores in all contexts.

In total, 193 adult individuals had at least one observable first molar, and 606 adult molars were observed for severity of attrition. No evidence for activity-related wear was observed. Overall, males from both mass graves had the highest attrition scores, while males and females from the GC, as well as females from MG2 had the lowest (Fig 4). The distribution of scores was not substantially different between females from all contexts (p = 0.475) but it was significantly different in young males (p = 0.013). Attrition scores also proved to be significantly different between older males and females from MG1 (p = 0.028) and young and older males and females from MG2 (p = 0.007 and p = 0.004, respectively).

Seventy-two non-adults had at least one observable M1 (25 from the GC, 32 from MG1 and 15 from MG2). The average M1 attrition scores were similar for MG2 and the GC, while individuals from MG1 had the highest scores (Fig 4). Attrition proved to be significantly higher in MG1 than in both, the GC (p = 0.015) and MG2 (p = 0.037). In total, 254 first molars were observed in non-adults.

#### Caries, periapical lesions, periodontal disease, and AMTL

Caries could be observed in 225 adult individuals who had at least one tooth. All observed individuals and teeth/alveoli/quadrants for these conditions can be seen in Table 2. Overall, caries rates were above 50% in all groups, except in females from the GC. Males from the GC had the highest caries rates overall (61.4%), while females from this context had the lowest (40.0%). The difference, however, was not statistically significant (p = 0.082). Caries prevalence rates in the sex groups between the contexts were not substantially different.

**Table 2:** Caries, periapical lesions, periodontal disease and AMTL in the adult population by individual, and by tooth/alveolus/quadrant count.

	*By individual*				*By tooth/alveolus/quadrant*			
	F	%	M	%	F	%	M	%
	**Caries**							
*GC*	16/40	40.0	27/44	61.4	41/693	5.9	72/921	7.8
*MG1*	17/29	58.6	26/52	50.0	48/601	8.0	56/1114	5.0
*MG2*	14/25	56.0	19/35	54.3	31/513	6.0	48/750	6.4
	**Periapical lesions**							
*GC*	14/40	35.0	18/44	40.9	24/1014	2.4	42/1285	3.3
*MG1*	10/29	34.5	19/52	36.5	24/847	2.8	40/1518	2.6
*MG2*	7/25	28.0	20/36	55.6	18/744	2.4	38/1032	3.7
	**Periodontal disease**							
*GC*	11/40	27.5	18/44	40.9	52/265	19.6	85/326	26.1
*MG1*	10/29	34.5	20/52	38.5	45/220	20.5	120/405	29.6
*MG2*	9/25	36.0	16/36	44.4	47/189	24.9	61/270	22.6
	**AMTL**							
*GC*	25/40	62.5	23/44	52.3	135/1014	13.3	137/1285	10.7
*MG1*	17/29	58.6	31/52	59.6	77/847	9.1	127/1518	8.4
*MG2*	16/25	64.0	21/36	58.3	108/744	14.5	87/1032	8.4

F-females; M-males; GC-general cemetery; MG1, MG2-mass graves; AMTL-ante-mortem tooth loss

The highest prevalence of periapical lesions in adults was observed in males from MG2 (55.6%), while the lowest prevalence was observed in females from this context (28.0%). The difference was not statistically significant between these groups (p = 0.062), and the lesions were also equally distributed between sex groups from the other contexts. Accordingly, the prevalence rates of periapical lesions were not consistent with those of caries, discussed above.

Periodontal disease did not show substantial differences between same sex groups from different contexts. Likewise, the differences between sex groups in the same contexts were not pronounced. Males from MG2 had the highest prevalence of the condition (44.4%), while females from the GC had the lowest (27.5%) (Table 2).

Ante-mortem tooth loss affected over 50% of people from all contexts, although the prevalence was somewhat higher in females from the GC and MG2 (62.5% and 64.0%, respectively). There were no pronounced differences in prevalence rates either between, or within, the contexts (Table 2).

In the non-adult population, there were low caries rates in both deciduous and permanent teeth (Table 3). Overall, 20 of 158 individuals (12.6%) with at least one erupted deciduous and/or permanent tooth were affected. There were no individuals with caries affecting both permanent and deciduous dentition.

**Table 3:** Caries in the non-adult population by individual and by tooth count.

	*By individual*						*By tooth*					
	D	%	P	%	Total	Total %	D	%	P	%	Total	Total %
*GC*	3/84	3.6	4/38	10.5	7/98	7.1	6/744	0.8	5/376	1.3	11/1120	2.1
*MG1*	2/22	9.1	6/35	17.1	8/40	20.0	3/150	2.0	11/630	1.7	14/780	3.7
*MG2*	3/20	15.0	2/19	10.5	5/26	19.2	5/171	2.9	2/302	0.7	7/473	3.6

D-deciduous; P-permanent

The lowest overall (deciduous and permanent) caries rates were in the children from the GC (7.1%), while in MG1 and MG2 they were substantially higher (20.0% and 19.2%, respectively). The difference between caries rates in the GC, MG1 and MG2 was not statistically significant (p = 0.054). The youngest individuals with caries in the deciduous dentition were 4–5 years old (individuals GC 280 and MG1 572). In both, deciduous molars were affected.

Only one non-adult individual had a periapical lesion (burial 340, 17–19 years old, from MG1). It affected the root of the right mandibular M1, which had been lost ante-mortem. Periodontal disease and AMTL were not observed in children.

#### Calculus

**Fig 5:**
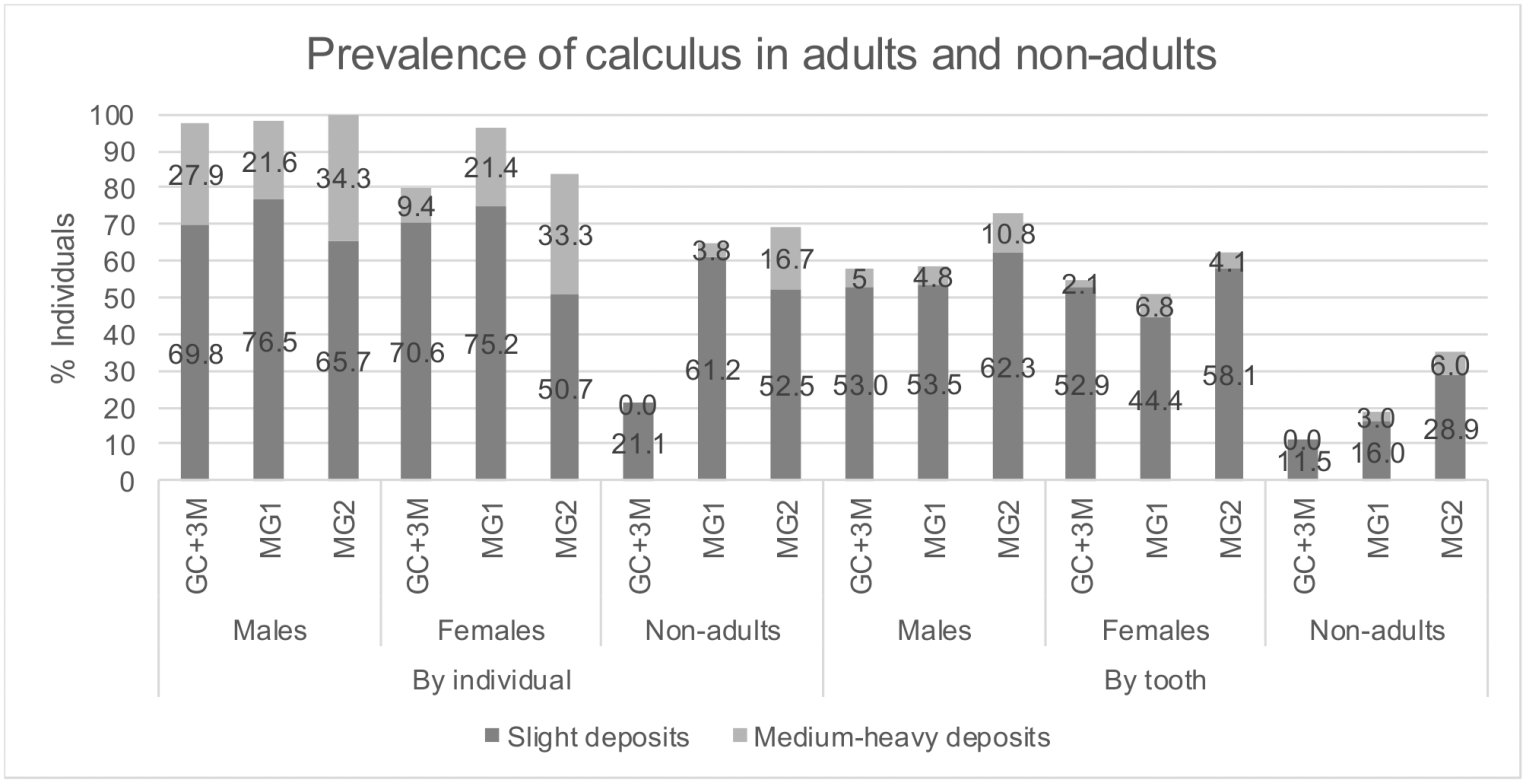
Prevalence of medium-severe calculus deposits in adults and children. Only total (deciduous and permanent) prevalence is given in this figure for calculus in children.

In the adult population, calculus deposits were present on the dentitions of most adults (Fig 5). The lesions affected slightly fewer females than males, especially with regard to the GC and MG2. In the non-adult population, calculus deposits affected individuals with both permanent and deciduous teeth. In total, most individuals affected derive from MG2 (18 of 26, or 69.2%) followed by MG1 (26 of 40, or 65.0%), but there were only 21.1% children (20 of 95) with calculus deposits on the permanent and/or deciduous teeth in the GC. The difference between the GC and MG1 and the GC and MG2 was statistically significant (both p<0.001). Individuals from MG1 and MG2 had a relatively high prevalence of dental calculus on their permanent dentitions (24 of 40, or 60.0% and 13 of 26, or 50.0%, respectively), while only 13 of 95, or 13.7% of individuals were affected in the GC. With regard to calculus deposits on deciduous teeth, the highest prevalence rates were in individuals from MG2 (nine of 26, or 34.6%), while fewer non-adults were affected from MG1 and the GC (four of 40, or 10.0%, and nine of 95, or 9.5%, respectively).

There were no children with medium-severe calculus deposits from the GC, affecting either deciduous or permanent dentitions. In MG1, one individual had heavier deposits on both deciduous and permanent teeth (Individual 490 from MG1, aged 15–16 years at death). In MG2, although no individuals with deciduous teeth had heavier calculus deposits, there were three non-adults with permanent dentitions affected.

Analysis of the amount of calculus revealed more differences in adults between contexts, than the total prevalence. Males from MG2 proved to have the highest prevalence of medium-severe lesions in this sex group, and overall (12 of 35, or 34.3%), while only 9.4% (three of 32) of females from the GC had heavier calculus deposits, compared to over 20% in females from the other two contexts (six of 28 and seven of 21 in MG1 and MG2, respectively). The difference was statistically significant between females from the GC and MG2 (p = 0.038), but not between the GC and MG1 (p = 0.281).

The number of observed individuals and teeth for this analysis is given for adults in S3 Table and for non-adults in S4 Table.

#### Results of isotope analysis

**Fig 6:**
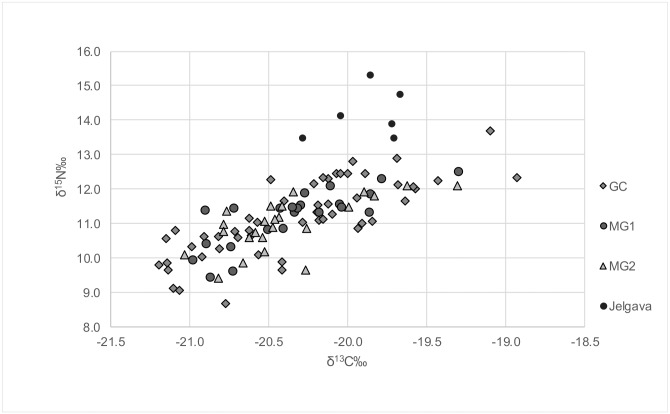
δ^13^C and δ^15^N values in the St Gertrude and Jelgava populations.

The range of δ^15^N values in the St Gertrude’s cemetery was between 8.7‰ and 13.7‰ (both values from individuals from the GC), and the range of δ^13^C values was between -21.2‰ and -18.9‰ (also both from the GC). In Jelgava, δ^15^N values ranged between 13.5‰ and 15.3‰, and δ^13^C values between -20.3‰ and -19.7‰. Detailed measurement data for isotope analysis can be observed in S5 Table. Results of carbon and nitrogen isotope analyses revealed a clear division in δ^15^N values between the populations of Jelgava and St Gertrude (Fig 6).

Most individuals had δ^15^N values below 13.0‰, except individual 92 (male, 45–55 years old, from the GC). In the Jelgava population, however, all δ^15^N values were above 13.0‰.

As seen in Fig 6, δ^13^C and δ^15^N values in St Gertrude population correlate positively (both increase and/or decrease in concert). Linear correlation analysis resulted in r = 0.8.

When the individuals from the cemetery were divided into males and females, a number of patterns in the δ^13^C and δ^15^N value distributions emerged. Males from the GC had higher mean δ^15^N and δ^13^C values than females from the same context (Table 4). In MG2, while males had higher δ^13^C values, δ^15^N values were higher in females. In MG1, males had lower δ^13^C and δ^15^N values than females, and the lowest average δ^13^C values in the population. Females from MG1 had the highest δ^15^N values.

**Table 4:** Mean δ^15^N and δ^13^C values in the adults and children from Jelgava.

*Context*	*Males*					*Females*				
	n	δ^13^C	SD	δ^15^N	SD	n	δ^13^C	SD	δ^15^N	SD
GC	31	-**20.1**	0.55	**11.3**	1.17	20	-**20.6**	0.45	**10.9**	0.90
MG1	10	-**20.5**	0.49	**11.1**	0.89	11	-**20.2**	0.32	**11.4**	0.76
MG2	15	-**20.4**	0.37	**10.9**	0.74	8	-**20.4**	0.49	**11.1**	0.85
Jelgava children										
	6	-**19.9**	0.24	**14.2**	0.73					

n-number of individuals; SD-standard deviation

There were no statistically significant differences in the distribution of δ^15^N values between males and females from all contexts (p = 0.257 and p = 0.243, respectively). Likewise, the differences in δ^15^N values between sex groups from the same contexts were not statistically significant (p = 0.078 (GC), p = 0.379 (MG1) and p = 0.401 (MG2)). The differences in δ^13^C values, however, were more pronounced. Although the distribution was not significantly different in males from all contexts (p = 0.101), δ^13^C values proved significantly higher in females from MG1 than females from the GC (p = 0.012). δ^13^C values did not significantly differ between females from the GC and MG2 (p = 0.390) and females from both mass graves (p = 0.126). With regard to differences between the sexes in the same contexts, δ^13^C values were significantly higher in males than females from the GC (p = 0.005), and significantly lower in males than females from MG1 (p = 0.037). The differences between males and females from MG2 were not statistically significant (p = 0.772).

## Discussion

### Variation in diet as expressed by oral health indicators

The first differences between the three contexts emerged when comparing dental attrition rates, indicating that the relative coarseness of the diet varied between males, females and children. The observed attrition rates were significantly lower in young males from the GC than young males from both mass graves, and significantly lower in females than males from both mass graves. This was also partly the case in non-adult individuals, with attrition rates significantly lower in the GC and MG2 than MG1. This suggests that young males from both mass graves, and children from MG1, had a coarser diet than most other people buried in the cemetery. Although bread was a staple food in post-medieval Latvia [9], its coarseness differed across regions and/or individual populations, resulting from either different milling techniques (by hand, or in a wind or water powered mill) or inclusion of other plant material in the flour, or both. For example, according to historical sources, peasants in some regions had hand mills and made their own flour, and the resulting flour had a high proportion of bran, as well as bits of straw, mixed in with it [105]. There are also accounts of various wild grasses, as well as moss, being added to bread in years of poor harvest [106, 107]. On the other hand, the flour ground in mills for the upper classes was finely sieved, thus leaving out bran and other particles [10]. Moreover, cereal grains were used in different meals, including porridge, and not just in bread [10, 108, 109], and they might have been cooked differently in some population groups according to the age and/or sex of the people. This might have been the case for most males, females and children buried in MG2. The observed differences in dental attrition rates are in support of hypotheses one and two, and suggest that different population groups are present in all three contexts. A more detailed study including the attrition of all types of teeth would be necessary to further explore the findings of this study.

With regard to caries in adults and non-adults, there were no significant differences in the same sex groups between the contexts, despite the varied attrition rates discussed above. It can therefore be concluded that in the overall population, attrition was not linked to caries prevalence. One of the possible explanations for the inconsistent results might be more abrasive particles in the diet of some groups, as discussed above, rather than a smaller proportion of carbohydrates.

The comparatively lower caries rates in females from the GC are difficult to explain. If these individuals had a higher proportion of products which are known to prevent the disease, such as milk, cheese, or sea food in their diet compared to males [23, 39], then this would likely be expressed in higher δ^13^C and/or δ^15^N values. This, however, was not the case (see the following section).

The highest prevalence of periapical lesions in males from MG2 seemed to be linked to dental attrition, rather than caries. As detailed above, this can be explained by severe attrition, whereby the dental pulp cavity is exposed and thus becomes infected [40]. In males from the GC, however, the high prevalence of periapical lesions did seem to be linked to high caries rates, whereby the pulp became exposed because of advanced dental decay. However, a higher prevalence rate might have been detected in all groups if radiographic analysis had been carried out.

With regard to periodontal disease and AMTL, it seems inconsistent that males from MG2 had the highest rates of periodontal disease, despite having the highest attrition rates. As mentioned above, periodontal disease is often associated with softer diets. Since the condition was recorded by observing the alveolar margin, rather than the length of the exposed root, this discrepancy in frequency rates is unlikely to have occurred because of attrition initiated continuous eruption [42]. It is therefore likely that periodontal disease in groups with higher attrition rates was the result of bacterial activity in the dental plaque, regardless of a coarser diet [39, 44, 110]. It is also possible that psychological stress was an additional factor in the development of the disease in males from MG2 [47, 48]. This, however, would be difficult to test in an archaeological population. In males from the GC, however, the low attrition is consistent with a high prevalence of periodontal disease. Given the high rates of destructive dental diseases in the population discussed above, the over 50% AMTL prevalence in all contexts and sex groups does not seem unusual.

While the lack of statistically significant differences in the prevalence of caries, periapical lesions, periodontal disease and AMTL between any of the demographic groups in this study supports hypothesis three, it can be argued that similarly high amounts of carbohydrates, as discussed above, contributed to relatively equal rates of the four conditions in all contexts. As shown by significant differences in dental attrition rates, however, the main differences in diet between the groups seem to have been based on its coarseness, as discussed above.

Moving on to calculus, the significant differences in the prevalence of medium-severe deposits between females from the GC and MG2, as well as the total prevalence between children from the GC and both mass graves, might indicate a difference with regard to the amount and/or composition of dietary carbohydrates in these groups. Chemical analysis of the calculus deposits would be necessary to confirm any differences in the composition of dietary carbohydrates between the people buried in different contexts. On the other hand, recent clinical studies suggest that certain amino-acids, which are naturally present in red meat, poultry and milk, have a potential to remove dental plaque, if used in concentrated amounts [52, 53]. If considerable dietary intake of such animal protein did have any impact on lower prevalence of plaque, it would be expressed in higher δ^15^N values. This was not the case in St Gertrude’s cemetery population, whereby no statistically significant differences in δ^15^N values were observed between the groups (see the following section). Alternatively, the observed differences in heavy calculus deposits might be a result of differential survival in the burial environment [111], or differential presence and severity on particular teeth, which might be related to teeth having been lost ante- or post-mortem. A more detailed analysis of dental calculus by the number and type of affected teeth would be necessary to further explore the differences.

In summary, the differences in oral health indicators discussed above provide sufficient evidence to support hypotheses one and two, that people buried in the GC and mass graves represent three different population groups, even though the prevalence of destructive dental disease did not prove to be statistically significant between them.

While it is likely that the populations were different, it is not possible to confirm by dental analysis alone whether either of the populations from the mass graves comprised rural immigrants. A recent study comparing dental caries, AMTL and periapical lesions in post-medieval cemetery populations in Latvia found that there were no particular patterns for rural and urban, as well as high and low status populations [28]. The lack of a pattern was explained by the different political situations in the observed regions; Latvia was divided between Sweden and the Polish-Lithuanian Commonwealth during the period in question, with land mainly in the hands of German landowners. The possible differences between cultural beliefs, fertility demands, availability of food and the general quality of life were also suggested as causes. The study also found that there were substantial differences even between two neighbouring rural cemetery populations in Vidzeme, likely due to different diet. With regard to comparative studies from other Baltic countries, two contemporary populations from Estonia (rural Tääksi, and urban Pärnu St John’s Church cemetery) both yielded very similar rates of caries and periapical lesions as observed in the St Gertrude’s cemetery population [112, 113]. The lack of similar patterns in dental disease between urban and rural populations from Latvia, and the scarcity of data from other post-medieval Baltic populations, made a regional comparison with the St Gertrude’s cemetery difficult.

### Dietary differences as expressed by isotope analysis

As shown in Fig 6, the population of Jelgava had substantially higher δ^15^N values than St Gertrude. Geographically, the city of Jelgava is situated inland, adjacent to the River Lielupe (Fig 1). The high δ^15^N values in this population may therefore be explained by the presence of fish from the River Lielupe in their diet. Alternatively, some influence of breastfeeding is also possible in the three younger children (below two years of age) from this population, since during breastfeeding, δ^15^N values of infants have been shown to be 2–3‰ higher compared to their mothers or wet-nurses in modern and archaeological populations [114–116]. Ethnographic and historical evidence suggests that most children in past Latvian populations were fully weaned by the age of two years [117]. This would mean that the δ^15^N values in older children would be closer to those of the adult population. In the absence of adult data from the Jelgava population, it is hypothesised that the δ^15^N values above 13.0‰ in all children, compared to St Gertrude’s population, are more likely to be the result of a higher proportion of freshwater fish in the diet, rather than breastfeeding, although a further study is necessary to fully test this hypothesis. The Jelgava population was used in this study in order to explore obvious differences between two contemporary cemetery sites, and thus to aid discussion of the possible presence of populations of different origins who were buried in St Gertrude’s cemetery. This first step to comparative analysis pointed to substantial dietary differences between the Jelgava and St Gertrude populations, but no differences between the groups within St Gertrude’s cemetery. However, the region of Vidzeme from which the rural migrants are believed to have derived [1, 3, 4] has a long coastline. Consequently, populations living close to the coast might well be isotopically indistinguishable from those living in Riga.

The positive correlation coefficient for δ^13^C and δ^15^N values in people from St Gertrude cemetery suggests that the observed values were mainly influenced by a marine component in their diet, rather than differential terrestrial δ^13^C sources. The lack of a consistent pattern by context, however, is suggestive of high individual variability in the amount of marine protein in the diet of the whole population. Nevertheless, small differences in δ^13^C and δ^15^N values for the people buried in St Gertrude’s cemetery became apparent when they were analysed by sex.

The δ^15^N values were not significantly different between the contexts, indicating that most people had similar amounts of protein in their diet. With regard to significant differences observed between δ^13^C values in females from MG1 and the GC, and between the sex groups in these two contexts, the absolute mean δ^13^C values in all groups only differed by 0.4‰. This amount is too small to suggest meaningful dietary differences between any groups, with regard to the marine component, as was also supported by the lack of significant differences in δ^15^N values. Indeed, differences in δ^13^C values might also be caused by small temporal variations in the local terrestrial δ^13^C, which are influenced by environmental factors such as solar radiation, temperature, and moisture [118–120].

The apparent lack of freshwater resources in the diet of the population is surprising, considering that they lived by a large river, and that freshwater fish were routinely caught by the local fishermen to be sold in the markets [121]. It seems that instead, the population relied to a far greater extent on fish from the sea. Historical evidence suggests that salted fish was not only readily sold in Riga, but also transported to inland markets [10]. The good storage properties of salted fish might have made them cheaper than fresh fish, and thus perhaps more available to the local population.

At the beginning of this study it was expected that dietary isotope analysis would be consistent with specific destructive dental diseases, and caries in particular. As discussed above, marine protein, similar to certain milk products, can prevent the development of dental caries [23], and a correlation between a high proportion of marine fish in the diet and low caries rates has been found in other archaeological studies [23, 39, 122]. In this study, however, despite the significant differences observed in δ^13^C values between some groups, meaningful differences in the amount of marine protein in the diet could not be detected. It is possible that the proportion of marine resources in the diet of this population was too low to have any impact on dental decay, especially if carbohydrates were a dietary staple, as discussed above. This is also supported by the mean δ^13^C values of all groups being above -20.0‰, close to terrestrial end-point in diet (-21.0‰), and with no individual values exceeding -19.0‰. Likewise, the prevalence of dental diseases and the dietary carbon and nitrogen values observed in St Gertrude’s cemetery are consistent with data from Roman Winchester, whereby relatively high caries rates in the population were also explained by a diet based predominantly on carbohydrates [123].

## Conclusion

The main aim of this paper was to explore if there were different population groups buried in the St Gertrude’s cemetery, based on differences in the prevalence of dental pathological conditions and attrition and dietary stable isotope values.

Firstly, there were differences between contexts with regard to dental attrition and calculus deposits. Although most contrasts were observed between mass graves and the GC, there was also evidence for differences between the mass graves, mainly expressed by significantly higher attrition rates in children from MG1 than in both other contexts. This evidence supports the possible presence of more than one population group in the mass graves, albeit with more similarities between them when compared to Gertrude villagers buried in the GC. The lack of significant differences in destructive dental disease between any of the groups is suggestive of a similar amount of carbohydrates in their diet.

The results of the dietary stable isotope analysis, however, did not support evidence for the presence of different populations when compared by context. The individuals from all three groups were spread across the observed range for both δ^13^C and δ^15^N. The observed variability of δ^13^C values suggested that all groups had equal access to marine fish and/or other marine protein, but that it was accessed differentially among individuals, without clear evidence for any pattern by context and/or sex groups. Likewise, no significant differences were found in δ^15^N values between any groups. The low mean and individual δ^13^C values also suggest that the marine input in the diet of the whole population might have been too low to have any preventative effect on destructive dental disease.

The three hypotheses of this study were only partly supported, because evidence for several population groups being present in the cemetery was not fully supported by all observed variables. However, as discussed above, it is possible that local populations living in close proximity to the sea, and/or inland with regular access to salted fish, might be isotopically indistinguishable. Further research is ongoing to look at strontium isotope data from selected individuals in each context, which might help to identify any migrants, and thus provide further evidence about the population groups buried in St Gertrude’s cemetery. Furthermore, aspects of general health, including indicators of interrupted growth in childhood, will also be compared between the contexts.

Currently, there is a lack of similar studies from the region, hampering comparisons with other contemporary coastal and/or inland populations, especially with regard to dietary isotope values. This study is therefore an important addition to the published literature, having generated comparable and detailed data on oral health indicators, as well as a set of much needed reference dietary isotope values, which can be readily used in the future by researchers all over the Baltic region.

## Supporting information

S1 Table Prevalence of caries, periapical lesions, periodontal disease and AMTL by affected/observed individuals and tooth/quadrant/alveolus count in young and older adults. (PDF)

S2 Table Results of statistical analysis for dental attrition, dental disease, and isotope analysis. (PDF)

S3 Table Prevalence of calculus deposits in adult individuals by affected/observed individual/tooth count. (PDF)

S4 Table Prevalence of calculus deposits in non-adult individuals by affected/observed individual/tooth count. (PDF)

S5 Table Details of measurement data for δ^15^N and δ^13^C isotopes. (PDF)
