# Supplementary material for: Dental disease and dietary isotopes of individuals from St Gertrude Church cemetery, Riga, Latvia
Source: PLoS One. 2018 Jan 24;13(1):e0191757. doi: 10.1371/journal.pone.0191757 (PMC5783410; doi:10.1371/journal.pone.0191757)
Supplement: S1 Table — (PDF) [file pone.0191757.s001.pdf]

S1 Table. Prevalence of caries, periapical lesions, periodontal disease and AMTL by affected/observed individuals and tooth/quadrant/alveolus count in young and older adults.

	By individual				By tooth			
	Young		Older		Young		Older	
	Males	Females	Males	Females	Males	Females	Males	Females
	**Caries**							
**GC**	8/19	2/14	19/25	14/26	13/457	7/280	59/464	34/413
**MG1**	8/18	8/16	18/34	9/13	16/403	13/348	40/711	35/253
**MG2**	4/13	4/8	15/22	10/17	7/308	5/206	41/442	26/307
	**Periapical lesions**							
**GC**	2/19	2/14	16/25	12/26	2/546	2/346	40/739	22/668
**MG1**	3/18	1/16	16/34	9/13	3/517	1/439	37/1001	23/408
**MG2**	2/13	1/8	18/23	6/17	4/369	1/246	34/663	17/498
	**Periodontal disease**							
**GC**	3/19	2/14	15/25	9/26	10/143	10/93	75/183	42/172
**MG1**	2/18	3/16	18/34	7/13	7/138	13/116	113/267	32/104
**MG2**	4/13	1/8	12/23	8/17	11/99	8/64	50/171	39/125
	**AMTL**							
**GC**	3/19	5/14	20/25	20/26	4/546	7/346	133/739	128/668
**MG1**	4/18	5/16	27/34	12/13	6/517	6/439	121/1001	71/408
**MG2**	4/13	2/8	17/23	14/17	7/369	4/246	80/663	104/498
