# Supplementary material for: Dental disease and dietary isotopes of individuals from St Gertrude Church cemetery, Riga, Latvia
Source: PLoS One. 2018 Jan 24;13(1):e0191757. doi: 10.1371/journal.pone.0191757 (PMC5783410; doi:10.1371/journal.pone.0191757)
Supplement: S2 Table — (PDF) [file pone.0191757.s002.pdf]

S2 Table. Results of statistical analysis for dental attrition, dental disease, and isotope analysis.

Groups tested	Context	Test used	Statistics	P value
**Dental attrition**				
**Females**	GC, MG1, MG2	Kruskal-Wallis	N=79, H=1.49, df=2	p=0.475
**Males**			N=115, H=10.94, df=2	p=0.004*
**Young males**	GC, MG1, MG2	Kruskal-Wallis	N=49, H=8.62, df=2	p=0.013*
**Young males**	GC, MG1	Mann-Whitney^	N=37, U= 237.5, z=-2.01	p=0.044*
	GC, MG2		N=31, U=180, z=-2.66	p=0.008*
**Older males**	GC, MG1, MG2	Kruskal-Wallis	N=66, H=2.69, df=2	p=0.2605
**Males, females**	GC	Mann-Whitney	N=69, U=585.5, z=0.07	p=0.944
	MG1		N=71, U=336.5, z=2.76	p=0.006*
	MG2		N=54, U=159, z=3.45	p=0.001*
**Young males, females**	MG1	Mann-Whitney^	N=33, U=124, z=0.38	p=0.704
	MG2		N=20, U=12.5, z=2.7	p=0.007*
**Older males, females**	MG1		N=38, U=66, z=2.2	p=0.028*
	MG2		N=34, U=59.5, z=2.86	p=0.004*
**Children**	GC, MG1, MG2	Kruskal-Wallis	N=72, H=7.59 df=2	p=0.022*
**Children**	MG1, GC	Mann-Whitney^	N=57, U=248.5, z=2.43	p=0.015*
	MG1, MG2		N=47, U=148.5, z=2.08	p=0.037*
**Caries**				
**Males, females**	GC	Chi-Square	N=84, X^2=3.02, df=1	p=0.082
**Children**	GC, MG1, MG2		N=164, X^2=5.81, df=2	p=0.054
**Periapical lesions**				
**Males, females**	MG2	Chi-Square	N=61 X^2=3.49, df=1	p=0.062
**Calculus deposits**				
**Females**	GC, MG1	Fisher Exact	N=60	p=0.281
	GC, MG2		N=53	p=0.038*
**Children**	GC, MG1	Chi-Square	N=135; X^2=22.28, df=1	p<0.001*
	GC, MG2		N=121 X^2=19.82, df=1	p<0.001*
**Isotope analysis**				
**δ^{15}N**				
**Males**	GC, MG1, MG2	Kruskal-Wallis	N=56, H=2.72, df=2	p=0.257
**Females**			N=39, H=2.83, df=2	p=0.243
**Males, Females**	GC	Mann-Whitney	N=51, U=218.5, z=1.76	p=0.078
	MG1		N=21, U=42, z=0.88	p=0.379
	MG2		N=23, U=73.5, z=-0.84	p=0.401
**δ^{13}C**				
**Males**	GC, MG1, MG2	Kruskal-Wallis	N=56, H=4.6, df=2	p=0.101

**Females**	GC, MG1, MG2	Kruskal-Wallis	N=39, H=6.93, df=2	p=0.031*
	GC, MG1	Mann-Whitney^	N=31, U=171.5, z=-2.52	p=0.012*
	GC, MG2		N=28, U=97.5, z=-0.86	p=0.390
	MG1, MG2		N=19, U=25, z=1.53	p=0.126
**Males, Females**	GC	Mann-Whitney	N=51, U=165, z=2.79	p=0.005*
	MG1		N=21, U=25, z=2.08	p=0.037*
	MG2		N=23, U=55, z=0.29	p=0.772

*-statistically significant difference; ^-post-hoc test
