# Supplementary material for: Dental disease and dietary isotopes of individuals from St Gertrude Church cemetery, Riga, Latvia
Source: PLoS One. 2018 Jan 24;13(1):e0191757. doi: 10.1371/journal.pone.0191757 (PMC5783410; doi:10.1371/journal.pone.0191757)
Supplement: S3 Table — (PDF) [file pone.0191757.s003.pdf]

S3 Table. Prevalence of calculus deposits in adult individuals by affected/observed individual/tooth count.

By individual						
	Males			Females		
	**GC**	**MG1**	**MG2**	**GC+3M**	**MG1**	**MG2**
Calc	43/44	51/52	35/35	32/40	28/29	21/25
Calc2	12/43	11/51	12/35	3/32	6/28	7/21
**By tooth**						
Calc	535/922	650/1114	548/750	381/693	308/601	319/513
Calc2	27/535	31/650	59/548	8/381	21/308	13/319

Calc – calculus present; Calc2 – medium-heavy deposits
