# Supplementary material for: Dental disease and dietary isotopes of individuals from St Gertrude Church cemetery, Riga, Latvia
Source: PLoS One. 2018 Jan 24;13(1):e0191757. doi: 10.1371/journal.pone.0191757 (PMC5783410; doi:10.1371/journal.pone.0191757)
Supplement: S4 Table — (PDF) [file pone.0191757.s004.pdf]

S4 Table. Prevalence of calculus deposits in non-adult individuals by affected/observed individual/tooth count.

By individual									
	Deciduous/total			Permanent/total			Total		
	GC	MG1	MG2	GC	MG1	MG2	GC	MG1	MG2
Calc	9/95	4/40	9/26	13/95	24/40	13/26	20/95	26/40	18/26
Calc2	0	1/4	0	0/13	1/24	3/13	0/20	1/26	3/18
**By tooth**									
Calc	31/744	7/150	32/171	98/376	140/630	132/302	129/1120	148/780	165/473
Calc2	0/31	1/7	0/32	0/98	3/140	6/132	0/129	4/148	6/165

Calc – calculus present; Calc2 – medium-heavy deposits
