# Supplementary material for: Dental disease and dietary isotopes of individuals from St Gertrude Church cemetery, Riga, Latvia
Source: PLoS One. 2018 Jan 24;13(1):e0191757. doi: 10.1371/journal.pone.0191757 (PMC5783410; doi:10.1371/journal.pone.0191757)
Supplement: S5 Table — (PDF) [file pone.0191757.s005.pdf]

S5 Table. Details of measurement data for δ^{15}N and δ^{13}C isotopes.

	Sample ID	Wt coll. mg	δ^{15}N ‰	Wt %N	δ^{13}C ‰	Wt %C	C/N atomic	% coll. yield
**1**	3M692 a	0.544	10.6	15.6	-20.7	42.6	3.19	19.3
**2**	3M692 b	0.575	10.6	16.0	-20.7	43.0	3.14	
**3**	3M697 a	0.562	10.3	15.9	-20.9	42.8	3.14	16.8
**4**	3M697 b	0.658	10.3	15.9	-20.8	42.8	3.14	
**5**	3M700 a	0.550	11.1	15.5	-20.6	42.4	3.20	15.7
**6**	3M700 b	0.554	11.2	15.5	-20.6	42.4	3.18	
**7**	3M701 a	0.572	9.7	15.8	-21.2	42.8	3.16	18.1
**8**	3M701 b	0.530	9.9	15.9	-21.2	42.8	3.13	
**9**	3M706 a	0.546	10.8	15.3	-20.8	42.7	3.25	17.2
**10**	3M706 b	0.770	10.7	15.7	-20.6	42.6	3.17	
**11**	K4 a	0.527	9.6	15.4	-20.4	41.7	3.16	12.7
**12**	K4 b	0.499	9.7	15.5	-20.4	42.0	3.16	
**13**	K5 a	0.529	11.5	15.5	-20.1	42.1	3.17	14.4
**14**	K5 b	0.509	11.1	15.4	-20.1	41.7	3.17	
**15**	K26 a	0.573	11.2	15.4	-20.2	42.1	3.18	14.1
**16**	K26 b	0.569	11.0	15.2	-20.2	41.8	3.20	
**17**	K30 a	0.561	12.4	15.4	-20.1	42.3	3.21	10.1
**18**	K30 b	0.612	12.3	15.3	-20.2	41.9	3.19	
**19**	K31 a	0.520	11.7	15.3	-19.8	42.8	3.26	10.3
**20**	K31 b	0.647	11.6	15.5	-19.5	42.4	3.19	
**21**	K37 a	0.523	10.9	15.3	-19.9	42.2	3.21	14.1
**22**	K37 b	0.611	10.8	15.6	-20.0	42.8	3.21	
**23**	K40 a	0.507	8.7	15.3	-20.7	41.5	3.16	10.9
**24**	K40 b	0.517	8.7	15.6	-20.8	42.2	3.16	
**25**	K43B a	0.668	9.1	15.6	-21.1	42.4	3.18	13.7
**26**	K43B b	0.504	9.0	15.4	-21.1	42.0	3.17	
**27**	K51 a	0.570	11.4	15.1	-20.3	42.4	3.29	15.7
**28**	K51 b	0.746	11.3	15.6	-20.1	42.3	3.16	
**29**	K53 a	0.543	12.5	15.4	-19.9	42.3	3.20	16.1
**30**	K53 b	0.635	12.4	15.5	-19.9	42.3	3.18	
**31**	K67 a	0.587	11.1	15.4	-19.9	42.3	3.20	8.0
**32**	K67 b	0.540	11.0	15.3	-19.9	42.1	3.22	
**33**	K69 a	0.643	11.1	3.3	-20.3	9.4	3.33	15.2
**34**	K69 b	0.566	11.3	15.5	-20.4	42.3	3.19	
**35**	K78 a	0.572	11.8	15.4	-20.0	42.4	3.21	18.3
**36**	K78 b	0.612	11.7	15.6	-19.9	42.4	3.16	
**37**	K87 a	0.620	12.8	15.7	-19.9	42.3	3.15	16.7
**38**	K87 b	0.572	12.8	15.6	-20.0	42.5	3.18	
**39**	K88 a	0.612	11.0	15.3	-20.3	41.9	3.19	9.0

**40**	K88 b	0.587	11.0	15.5	-20.3	42.1	3.17	
**41**	K92 a	0.551	13.7	15.2	-19.1	41.6	3.19	8.6
**42**	K92 b	0.549	13.7	15.5	-19.1	41.7	3.14	
**43**	K95 a	0.619	12.5	15.3	-20.0	41.6	3.18	11.2
**44**	K95 b	0.758	12.4	15.6	-20.1	42.4	3.17	
**45**	K95.2a*	0.602	12.3	15.2	-20.1	41.7	3.19	11.6
**46**	K95.2b*	0.717	12.3	15.3	-20.1	41.4	3.15	
**47**	K96 a	0.509	12.6	15.2	-20.0	41.9	3.21	9.4
**48**	K96 b	0.536	12.3	15.6	-20.0	42.5	3.17	
**49**	K100 a	0.614	9.9	15.5	-20.4	42.1	3.17	13.5
**50**	K100 b	0.571	9.8	15.6	-20.4	42.2	3.16	
**51**	K113 a	0.603	12.4	15.4	-20.0	41.8	3.18	10.9
**52**	K113 b	0.574	12.4	15.4	-20.1	41.9	3.18	
**53**	K114 a	0.687	10.4	15.2	-21.0	41.5	3.19	13.8
**54**	K114 b	0.790	10.3	15.5	-21.0	41.6	3.13	
**55**	K115 a	0.511	12.4	15.3	-18.9	42.2	3.21	14.6
**56**	K115 b	0.634	12.3	15.6	-18.9	42.3	3.16	
**57**	K135 a	0.602	9.7	15.5	-21.2	42.0	3.17	15.8
**58**	K135 b	0.594	9.6	15.6	-21.1	42.3	3.16	
**59**	K160 a	0.539	12.9	14.9	-19.7	41.3	3.23	13.5
**60**	K160 b	0.504	12.9	15.2	-19.7	41.9	3.21	
**61**	K187 a	0.556	12.2	15.4	-19.7	41.9	3.17	13.0
**62**	K187 b	0.601	12.1	15.6	-19.7	42.2	3.16	
**63**	K191 a	0.529	9.2	15.3	-21.0	42.1	3.21	9.3
**64**	K191 b	0.536	9.0	15.6	-21.2	42.6	3.19	
**65**	K234 a	0.563	11.1	15.4	-19.9	42.2	3.20	15.1
**66**	K234 b	0.609	11.1	15.9	-19.8	42.7	3.14	
**67**	K235 a	0.506	11.7	15.3	-20.1	41.6	3.17	8.5
**68**	K235 b	0.599	11.4	15.5	-20.2	41.8	3.15	
**69**	K262 a	0.556	11.1	15.6	-20.2	42.4	3.18	16.1
**70**	K262 b	0.580	11.1	15.9	-20.1	42.8	3.14	
**71**	K310 a	0.535	10.0	15.3	-20.6	41.9	3.19	12.4
**72**	K310 b	0.516	10.2	15.6	-20.6	42.1	3.14	
**73**	K321 a	0.501	12.4	15.0	-20.4	41.9	3.25	6.1
**74**	K321 b	0.599	12.2	15.3	-20.6	42.8	3.27	
**75**	K323 a	0.558	10.7	15.4	-20.8	41.9	3.17	6.7
**76**	K323 b	0.526	10.6	15.5	-20.8	41.9	3.16	
**77**	K342 a	0.522	12.0	15.3	-19.6	42.3	3.22	15.1
**78**	K342 b	0.551	12.0	15.6	-19.6	42.6	3.18	
**79**	K394 a	0.517	10.9	15.4	-20.5	42.0	3.18	12.6
**80**	K394 b	0.528	10.8	15.6	-20.5	42.4	3.17	

**81**	K410 a	0.506	10.7	15.3	-20.6	41.7	3.17	9.9
**82**	K410 b	0.508	10.7	15.5	-20.6	41.9	3.15	
**83**	K427 a	0.569	12.3	15.5	-19.4	42.3	3.19	9.6
**84**	K427 b	0.526	12.2	15.6	-19.5	42.2	3.16	
**85**	K462 a	0.501	10.9	15.4	-20.6	42.1	3.19	11.0
**86**	K462 b	0.516	10.8	15.7	-20.6	42.4	3.15	
**87**	K539 a	0.528	11.0	15.6	-20.6	42.9	3.21	11.8
**88**	K539 b	0.526	11.1	15.6	-20.6	42.4	3.16	
**89**	K557 a	0.559	11.7	15.5	-20.4	42.1	3.18	12.0
**90**	K557 b	0.505	11.6	15.5	-20.4	42.2	3.17	
**91**	K617 a	0.553	12.1	15.4	-19.5	41.9	3.17	10.1
**92**	K617 b	0.547	12.1	15.7	-19.7	42.3	3.15	
**93**	K631 a	0.570	10.6	15.5	-21.1	42.6	3.20	12.5
**94**	K631 b	0.671	10.6	15.2	-21.2	41.1	3.15	
**95**	K638 a	0.631	10.0	16.1	-20.9	43.4	3.15	15.0
**96**	K638 b	0.628	10.1	15.4	-20.9	41.1	3.12	
**97**	K639 a	0.753	10.0	15.5	-21.1	42.2	3.18	15.9
**98**	K639 b	0.771	9.8	15.4	-21.2	41.5	3.15	
**99**	K676 a	0.700	12.2	15.8	-20.2	42.6	3.15	10.2
**100**	K676 b	0.752	12.2	15.7	-20.2	41.9	3.12	
**101**	K677 a	0.606	10.6	15.4	-20.8	41.8	3.16	9.3
**102**	K677 b	0.596	10.6	15.1	-21.0	42.1	3.24	
**103**	K683 a	0.547	10.9	15.4	-21.1	42.7	3.23	6.9
**104**	K683 b	0.731	10.7	15.5	-21.1	42.1	3.16	
**105**	DA84 a	0.529	11.4	15.9	-21.0	43.9	3.22	11.7
**106**	DA84 b	0.553	11.4	15.8	-20.8	43.1	3.18	
**107**	DA84.2 a*	0.623	11.5	16.0	-20.7	43.4	3.15	11.6
**108**	DA84.2 b*	0.725	11.4	15.9	-20.7	43.0	3.15	
**109**	DA94 a	0.833	11.4	15.6	-20.5	42.5	3.19	5.8
**110**	DA94 b	0.639	11.3	15.5	-20.4	42.6	3.20	
**111**	DA110 a	0.634	10.8	15.7	-20.5	42.8	3.18	16.1
**112**	DA110 b	0.660	10.8	15.3	-20.5	41.2	3.14	
**113**	DA111 a	0.572	9.4	15.7	-20.8	43.0	3.17	13.9
**114**	DA111 b	0.520	9.4	15.6	-20.9	43.1	3.19	
**115**	DA112 a	0.524	11.5	15.7	-20.3	42.3	3.15	14.0
**116**	DA112 b	0.539	11.5	19.1	-20.3	51.4	3.13	
**117**	DA131 a	0.521	12.3	15.6	-19.8	42.4	3.16	9.8
**118**	DA131 b	0.656	12.3	12.1	-19.8	32.9	3.16	
**119**	DA139 a	0.522	12.6	19.0	-19.0	50.8	3.12	9.7
**120**	DA139 b	0.514	12.4	15.4	-19.6	41.2	3.13	
**121**	DA140 a	0.616	11.3	15.7	-20.3	42.4	3.14	16.1

**122**	DA140 b	0.503	11.3	18.4	-20.4	49.2	3.12	
**123**	DA143 a	0.552	11.5	15.6	-20.4	42.5	3.18	16.6
**124**	DA143 b	0.585	11.4	13.5	-20.5	37.1	3.20	
**125**	DA147 a	0.573	11.5	15.8	-20.3	42.7	3.16	15.7
**126**	DA147 b	0.510	11.4	17.2	-20.3	46.6	3.17	
**127**	DA153 a	0.701	9.9	15.8	-21.0	42.2	3.13	11.9
**128**	DA153 b	0.558	9.9	14.8	-21.0	39.8	3.13	
**129**	DA157 a	0.593	11.5	15.7	-20.3	42.6	3.16	11.3
**130**	DA157 b	0.527	11.4	20.6	-20.3	55.6	3.16	
**131**	DA163 a	0.564	11.9	15.6	-20.3	42.3	3.17	16.7
**132**	DA163 b	0.691	11.8	12.8	-20.3	34.5	3.14	
**133**	DA165 a	0.633	10.4	15.7	-20.9	42.2	3.14	11.9
**134**	DA165 b	0.566	10.4	17.3	-20.9	46.6	3.14	
**135**	DA182 a	0.522	10.7	15.6	-20.4	42.4	3.16	14.6
**136**	DA182 b	0.519	10.9	15.7	-20.4	42.1	3.14	
**137**	DA338 a	0.532	11.5	15.6	-20.1	42.2	3.15	10.8
**138**	DA338 b	0.525	11.6	16.0	-20.0	42.2	3.08	
**139**	DA488 a	0.538	10.2	15.6	-20.7	42.0	3.14	9.6
**140**	DA488 b	0.534	10.4	15.8	-20.7	42.1	3.11	
**141**	DA586 a	0.593	9.5	15.6	-20.7	42.2	3.16	12.9
**142**	DA586 b	0.570	9.7	15.5	-20.7	41.8	3.14	
**143**	DA622 a	0.511	11.3	15.6	-19.9	42.2	3.16	12.1
**144**	DA622 b	0.510	11.3	15.6	-19.9	41.8	3.13	
**145**	DA623 a	0.620	11.4	15.7	-20.1	42.2	3.14	10.8
**146**	DA623 b	0.600	11.5	15.8	-20.0	41.7	3.08	
**147**	DA623.2 a*	0.514	11.3	15.7	-20.1	42.4	3.15	16.8
**148**	DA623.2 b*	0.556	11.3	15.6	-20.3	42.3	3.16	
**149**	DA645 a	0.656	12.0	15.5	-20.1	42.3	3.18	15.9
**150**	DA645 b	0.520	12.2	15.7	-20.1	42.2	3.13	
**151**	DA658 a	0.617	11.9	16.3	-19.9	44.6	3.20	18.2
**152**	DA658 b	0.664	11.8	15.8	-19.8	42.2	3.12	
**153**	J15 a	0.668	15.3	16.3	-19.9	44.1	3.16	15.8
**154**	J15 b	0.583	15.4	16.0	-19.8	43.3	3.16	
**155**	J22 a	0.738	13.5	16.0	-20.2	43.2	3.14	20.6
**156**	J22 b	0.732	13.5	16.0	-20.4	43.8	3.19	
**157**	J33 a	0.685	14.7	16.1	-19.7	44.4	3.21	14.1
**158**	J33 b	0.620	14.8	15.8	-19.6	43.0	3.17	
**159**	J40 a	0.592	13.6	13.6	-20.6	42.5	3.65	2.9
**160**	J40 b	0.621	13.6	14.0	-20.4	42.5	3.55	
**161**	J95 a	0.653	14.0	15.8	-19.8	43.5	3.21	11.3
**162**	J95 b	0.700	13.9	15.8	-19.6	43.4	3.20	

**163**	J97 a	0.513	14.1	15.6	-20.1	43.9	3.28	8.6
**164**	J97 b	0.752	14.1	15.8	-20.0	43.5	3.21	
**165**	J98 a	0.639	13.5	16.2	-19.8	44.6	3.21	16.0
**166**	J98 b	0.579	13.5	16.0	-19.6	42.9	3.12	
**167**	ZR35 a	0.540	11.2	15.6	-20.5	42.9	3.20	14.5
**168**	ZR35 b	0.537	11.0	15.8	-20.4	42.4	3.14	
**169**	ZR42 a	0.587	10.6	15.4	-20.7	42.1	3.18	11.0
**170**	ZR42 b	0.533	10.6	15.5	-20.6	42.0	3.16	
**171**	ZR105 a	0.570	9.4	15.3	-20.9	42.4	3.24	10.8
**172**	ZR105 b	0.562	9.4	15.8	-20.8	42.8	3.16	
**173**	ZR118 a	0.514	10.9	15.4	-20.3	42.2	3.19	15.9
**174**	ZR118 b	0.523	10.8	15.8	-20.2	42.3	3.13	
**175**	ZR123 a	0.586	11.0	15.2	-20.9	42.9	3.28	10.4
**176**	ZR123 b	0.528	10.9	15.3	-20.6	42.4	3.23	
**177**	ZR159 a	0.571	10.8	15.7	-20.8	42.5	3.15	16.7
**178**	ZR159 b	0.518	10.7	15.6	-20.8	42.3	3.16	
**179**	ZR170 a	0.578	12.1	15.7	-19.3	42.3	3.14	17.7
**180**	ZR170 b	0.507	12.1	15.9	-19.3	42.6	3.13	
**181**	ZR197 a	0.538	11.2	15.8	-20.5	42.9	3.17	18.0
**182**	ZR197 b	0.532	11.1	15.7	-20.4	42.6	3.16	
**183**	ZR203 a	0.566	11.1	15.9	-20.5	43.4	3.19	13.9
**184**	ZR203 b	0.686	11.0	15.5	-20.6	42.1	3.18	
**185**	ZR204 a	0.766	11.5	16.0	-19.9	43.0	3.14	16.4
**186**	ZR204 b	0.755	11.4	15.3	-20.1	41.8	3.18	
**187**	ZR213 a	0.612	11.5	15.3	-20.3	41.9	3.20	3.3
**188**	ZR213 b	0.520	11.6	15.1	-20.6	42.7	3.29	
**189**	ZR214 a	0.738	10.9	15.4	-20.5	41.7	3.16	16.4
**190**	ZR214 b	0.777	10.9	16.3	-20.5	43.8	3.13	
**191**	ZR240 a	0.636	10.1	15.7	-21.1	43.0	3.19	15.7
**192**	ZR240 b	0.704	10.1	15.0	-21.0	40.7	3.16	
**193**	ZR241 a	0.732	11.8	15.9	-19.9	42.5	3.12	15.8
**194**	ZR241 b	0.516	11.8	15.7	-19.8	42.5	3.16	
**195**	ZR385 a	0.581	10.6	15.6	-20.5	42.5	3.18	15.7
**196**	ZR385 b	0.546	10.6	15.7	-20.6	42.5	3.17	
**197**	ZR444 a	0.528	10.2	15.6	-20.6	42.5	3.17	17.9
**198**	ZR444 b	0.573	10.2	15.7	-20.5	42.4	3.15	
**199**	ZR445 a	0.589	9.9	15.6	-20.7	42.4	3.18	15.6
**200**	ZR445 b	0.533	9.9	15.7	-20.6	42.5	3.16	
**201**	ZR469 a	0.591	10.7	15.7	-20.6	42.5	3.16	15.4
**202**	ZR469 b	0.590	10.7	15.6	-20.6	42.8	3.20	
**203**	ZR505 a	0.534	9.7	15.8	-20.2	42.5	3.14	19.0

**204**	ZR505 b	0.569	9.7	15.6	-20.3	41.8	3.13	
**205**	ZR511 a	0.573	12.1	15.7	-19.7	42.4	3.15	14.3
**206**	ZR511 b	0.601	12.1	15.8	-19.6	42.4	3.13	
**207**	ZR600 a	0.695	11.5	15.2	-20.4	41.4	3.18	11.0
**208**	ZR600 b	0.532	11.5	15.1	-20.6	41.6	3.22	
**209**	ZR600.2a*	0.717	11.3	14.9	-20.6	41.0	3.20	9.2
**210**	ZR600.2b*	0.617	11.4	14.9	-21.0	42.4	3.31	
**211**	ZR686 a	0.501	12.0	15.7	-19.9	42.7	3.18	17.5
**212**	ZR686 b	0.510	11.9	15.7	-19.9	42.4	3.16	
**213**	ZR698 a	0.561	12.0	15.8	-20.3	42.6	3.15	11.1
**214**	ZR698 b	0.626	11.9	15.8	-20.3	42.8	3.16	

*Control; 3M-burial pit; K-general cemetery; DA-mass grave 1; ZR-mass grave 2; J-Jelgava comparative population; a, b-duplicate measurements of the same sample
